# The Potential Proallergenic Activity of *Tranzschelia pruni-spinosae* and *Phragmidium rubi-idaei* in vitro Studies

**DOI:** 10.2147/JIR.S497219

**Published:** 2025-01-25

**Authors:** Monika Sztandera-Tymoczek, Sylwia Wdowiak-Wróbel, Urszula Świderska, Marta Palusińska-Szysz, Agnieszka Szuster-Ciesielska

**Affiliations:** 1 Department of Virology and Immunology, Institute of Biological Sciences, Maria Curie-Skłodowska University, Lublin, Poland; 2 Department of Genetics and Microbiology, Institute of Biological Sciences, Maria Curie-Skłodowska University, Lublin, Poland; 3 Department of Botany, Mycology and Ecology, Institute of Biological Sciences, Maria Curie-Skłodowska University, Lublin, Poland

**Keywords:** phytopathogenic microfungi, *Tranzschelia pruni-spinosae*, *Phragmidium rubi-idaei*, airway epithelial cells, inflammatory response

## Abstract

**Purpose:**

Allergic diseases have escalated to epidemic levels worldwide, impacting nearly 30% of the global population. Fungi are a significant source of allergens responsible for up to 6% of respiratory diseases in the general population. However, the specific cause of respiratory allergies often remains unidentified. This study aimed to investigate the potential of two common rust fungi, *Tranzschelia pruni-spinosae* and *Phragmidium rubi-idaei*, to trigger a proinflammatory response in vitro models representing the upper and lower respiratory tract.

**Materials and Methods:**

The BEAS-2B and A549 cell lines simulated upper and lower respiratory endothelial cells. The cytotoxicity of fungal extracts was evaluated using MTT and flow cytometry assays. Cell reactive oxygen species (ROS) production was measured via flow cytometry, while ELISA tests quantified the production of proinflammatory cytokines. Immunofluorescence techniques were employed to assess cell integrity markers.

**Results:**

Extracts from *T. pruni-spinosae* and *P. rubi-idaei* significantly stimulated the production of proinflammatory cytokines IL-1β and GM-CSF in both cell lines, all of which are associated with the development of allergic responses. The increase in these cytokines and the elevated ROS production were linked to the disruption of epithelial cell junctions.

**Conclusion:**

The findings suggest the potential of *T. pruni-spinosae* and *P. rubi-idaei* extracts to collectively disrupt the epithelial barrier in the upper and lower respiratory tract by inducing proinflammatory cytokines and the production of reactive oxygen species and metalloproteinases. Although none of the above parameters was spectacularly high, all of them together could cause a decrease in the presence of tight junction proteins, such as E-cadherin and occludin, in epithelial cells.

## Plain Language Summary

Phytopathogenic microfungi, commonly found in human surroundings, act as plant parasites. They adversely affect crop yields, lower the quality of agricultural products, and diminish the aesthetic value of ornamental plants. Furthermore, these fungi pose a potential health risk to humans, as they are major sources of allergens. The most notable allergenic fungi include *Alternaria, Aspergillus, Cladosporium, Penicillium*, and *Fusarium*. However, even widely distributed fungi like *T. pruni-spinosae* and *P. rubi-idaei*, which infect plums and raspberries, can trigger allergic reactions. These fungi heavily infest plants, releasing vast amounts of spores, particularly in the spring, summer, and fall. This increases the risk of human exposure to allergens as the plants and their parasitic fungi spread. Since standard skin or blood tests do not always pinpoint the root causes of allergies, identifying new fungal allergens is critical for more precise hypersensitivity diagnoses. Our research indicates that parasitic microfungi exhibit pro-inflammatory solid properties, which is the basis of allergenic reactions in in vitro upper and lower respiratory tract models.

## Introduction

The prevalence of allergic diseases is rapidly increasing worldwide and according to estimates by the World Allergy Organization (WAO), it varies from 10% to 40%, depending on the country. In most developed countries, allergy affects more than 20% of the population.^1^,^2^ While allergy was considered a rare disease at the beginning of the 20th century, the last few decades have dramatically increased its prevalence. According to research by the European Academy of Allergy & Clinical Immunology (EAACI), more than 150 million Europeans currently suffer from chronic allergic diseases, and 20% of them struggle with severe and debilitating forms of allergies. It is estimated that one in two Europeans will suffer from allergies by 2025. These figures are believed to be underestimated, as many patients do not report their symptoms or are misdiagnosed.^3^

The World Health Organization and the International Union of Immunological Societies (WHO/IUIS) currently indicate the existence of 750 allergens, including fungal allergens.^4^ Despite the extensive knowledge of the participation of fungi in the pathophysiology of allergic diseases, they still need to be sufficiently considered as allergens in basic research and clinical practice. However, this problem is confirmed by the fact that the incidence of respiratory allergies to fungi is estimated at 20–30% among atopic subjects and up to 6% in the general population.^5^ Another argument is that fungi are a more common cause of allergic reactions in the lower respiratory tract than pollen.^6^

Phytopathogenic microfungi, which are plant parasites, are commonly present in environments inhabited by humans. These fungi lead to widespread plant infestations, resulting in reduced crop yields, diminished quality of plant products, and loss of the aesthetic value of ornamental plants. The physiology and distribution of plants and fungi as well as the production of pollen and spores are influenced by such factors as geographical location, air quality, human activities, and local vegetation sources. Climate-related elements like temperature, humidity, and extreme weather also play a significant role.^7^,^8^

Conversely, phytopathogenic microfungi may pose a risk to human health since they are well-known and significant sources of allergens. Traditional skin or blood tests do not always successfully identify the specific allergen responsible for an allergy. The most significant allergenic fungi are typically found in the genera *Alternaria, Aspergillus, Cladosporium, Penicillium*, and *Fusarium*.^6^,^9^ However, it is possible that the highly prevalent native and invasive phytopathogenic microfungi responsible for widespread plant infestations could also act as sources of allergens. We propose that common phytopathogenic microfungi, which can be inhaled by humans, might be a potential trigger for allergic reactions.

In this study, we have presented the genetic identification and proinflammatory properties of two common rust species (Pucciniales): *T. pruni-spinosae*, which parasitizes *Prunus domestica*, and *P. rubi-idaei*, which infests *Rubus idaeus*. By using two cell model lines representing the upper (BEAS-2B) and lower (A549) respiratory tract cells, we demonstrate the proinflammatory activity of these microfungi and their ability to disrupt the epithelial barrier function in lung and bronchial cells. These processes are fundamental to the development of allergies and asthma. Thus, our research significantly enhances the understanding of new potential fungal allergens.

## Materials and Methods

### Plant Material and Morphological Identification

The fungi selected for the study are biotrophic organisms that cannot be grown on artificial substrates in the laboratory. Therefore, the research material was obtained from the natural environment. Plant organs affected by rust species (Pucciniales) were collected in Rymanów (Poland): *T. pruni-spinosae* on *Prunus domestica* L. on 11–12 Sept 2021 and 1−2 Oct 2021 (LBL M‒033122, leg. U. Świderska) and *P. rubi-idaei* on *Rubus idaeus* L. on 11–12 Sept 2021 and 1−2 Oct 2021 (LBL M‒033123, leg. U. Świderska). The host plants are commonly found in forests and thickets (*Rubus idaeus* L.) and cultivated by breeders (*Rubus idaeus* L., *Prunus domestica* L). They are not under any species protection and do not occur in protected areas.

The collected material, which included leaves and infecting fungi, was air-dried and stored in the herbarium at Maria Curie-Skłodowska University in Lublin (LBL). Initially, the morphological characteristics of the specimens were examined by mycologist U.Świderska through microscopic preparations stained with Lactophenol Cotton Blue dye. They were observed with an Olympus BX53 light microscope at magnifications of 40x, 100x, 400x, and 600x. Microphotographs of fungal diagnostic structures were captured using an Olympus digital camera SC180 attached to the microscope and an Olympus SZ10 stereoscopic microscope with an Olympus camera XC50. Further, the structures were coated with gold using an Emitech K550X Sputter Coater and visualized with a TESCAN Vega 3 LMU scanning electron microscope (from Brno, Czech Republic). Specimens containing the morphological features of the fungus, such as teliospores, were then prepared for additional laboratory analyses under the supervision of an Olympus SZ61 stereoscopic microscope. The samples were placed in test tubes and subsequently subjected to liquid nitrogen vapor for 24 hours before being ground to a powder using a mortar and pestle. The powdered fungal material was used to prepare crude extracts.

### Genetic Identification of Fungal Species

#### DNA Extraction

The DNA isolation from the fungal cells was performed using a DNeasy Plant Mini Kit (Qiagen) according to the manufacturer’s instruction. The purity and concentration of genomic DNA were checked by NanoDrop™ 2000/2000c measurements (Thermo Fisher Scientific, USA). The DNA was kept at –20 °C.

#### Molecular Phylogeny

The phylogenetic similarity of the studied fungi was assessed through a comparative analysis of the concatenated sequence of the internal transcribed spacer (ITS) region and large subunit (LSU) sequences of the rRNA gene. These sequences were amplified using primer pairs ITS5-u/ITS4-u and LR0R/LR6 (Vilgalys and Hester 1990; Pfunder et al 2001). The PCR amplifications were performed using the ReadyMix™ Taq PCR Reaction Mix kit (Sigma-Aldrich) following the manufacturer’s instructions. Each reaction mixture contained 50–100 ng of template DNA and 0.4 mm of each primer. In accordance with the established protocols, the PCR reactions were conducted in a thermocycler (TProfessional BASIC 96 Gradient, Biometra GmBH, Göttingen, Germany).^10^

The obtained amplicons were purified using a Clean-Up purification kit (A&A Biotechnology) and sequenced with a Terminator Cycle sequencing kit. The sequencing was performed on a 3500 Genetic Analyzer following the manufacturer’s instructions (Life Technologies). Using the BLAST tool, the resulting sequences were compared with those in the GenBank database. Sequence alignments were performed using ClustalX2^11^ and analyzed in the GeneDoc program.^12^ A phylogenetic tree was constructed from the generated sequences using the Neighbor-Joining (NJ) method in the MEGA11 program,^13^ with the Kimura two-parameter model as the nucleotide substitution model. The best-fitting evolutionary model for each gene was selected using jModelTest.^14^ The statistical significance of the tree was assessed with a bootstrap test (1000 replicates). The phylogenetic tree was visualized using the TreeView program.^15^

#### The GenBank Accession Numbers

The GenBank accession numbers of the analyzed sequences are as follows: LSU: OQ606766, ITS: OQ613349 for *Tranzschelia pruni-spinosae* and LSU: OQ606768, ITS: OQ613354 for *Phragmidium rubi-idaei*. The accession numbers of the reference strains are given on the phylograms.

#### Preparation of Crude Fungal Extracts

The fungal biomass was rinsed with acetone three times and dried for 24 hours at 37°C. The dried material was suspended in 0.05M Tris-HCl buffer at pH 8.0, at a ratio of 1 mL per 10 mg of dry weight, and subjected to three cycles of sonication (20 seconds of sonication followed by 2 minutes of cooling) in a room-temperature water bath (Elmasonic S100H, Elma, Germany). The extraction was performed overnight with shaking at 4°C, after which the extracts were centrifuged (804 × g, 10 minutes, 4°C). The supernatants were placed in a regenerated cellulose membrane with a molecular weight cut-off of 6–8 kDa (Spectrum Laboratories, Rancho Dominguez, CA, USA) and dialyzed for 24 hours at 4°C against 0.1M NH_4_HCO_3_ buffer at pH 8.4, with the buffer changed three times. The material was then lyophilized and resuspended in phosphate-buffered saline (PBS, Biomed, Poland). The protein concentration was determined using the Pierce™ BCA Protein Assay Kit (Thermo Fisher Scientific, Waltham, MA, USA) with bovine serum albumin as a standard.^16^ The crude fungal extracts prepared in three independent replicates were aliquoted into 100 µL portions and stored at −80°C for future analysis.

#### Cell Lines and Culture Media

The in vitro experiments used two adherent human airway cell lines: human alveolar epithelial cancer cells (A549, ATCC# CCL-185) and human normal bronchial epithelial cells (BEAS-2B, ATCC# CRL-9609). The A549 cells were cultured in a 1:1 (v/v) mixture of RPMI 1640 (Corning Media) and DMEM (Sigma-Aldrich) supplemented with 10% (v/v) heat-inactivated fetal bovine serum (FBS, EURx Molecular Biology Products) and 1% (v/v) penicillin-streptomycin (Sigma-Aldrich). The BEAS-2B cells were initially cultured in LHC-8 medium (Gibco) with 10% (v/v) heat-inactivated FBS and 1% (v/v) penicillin-streptomycin; after 24 hours, the medium was replaced with serum-free LHC-8 to maintain the characteristic cuboidal, polygonal morphology of respiratory epithelium.^17^ Both cell lines were incubated in standard conditions at 37°C, with 5% CO_2_ and 95% humidity. The cells were cultured until confluent and then passaged at a 1:3 ratio using PBS without Ca^2+^ and Mg^2+^ ions and 0.25% trypsin with 0.02% EDTA (Biological Industries).

#### Cytotoxicity study with the MTT assay

Cell viability was evaluated by assessing mitochondrial activity through the reduction of the yellow tetrazolium salt, 3-(4,5-dimethyl-2-thiazolyl)-2,5-diphenyl-2H-tetrazolium bromide (MTT), into purple formazan crystals. The intensity of the color, determined by spectrophotometric analysis of dissolved crystals, was indicative of the mitochondrial activity in living cells.^18^ In brief, the cells were seeded into 96-well plates (100 µL per well) at a density of 1x10^5^ cells/mL (the A549 cell line) or 2x10^5^ cells/mL (the BEAS-2B cell line) in a suitable culture medium with 10% FBS (Nunc, Roskilde, Denmark) and incubated at 37°C for 24 hours. After removing the medium, 100 µL of fungal extracts at varying concentrations (0.09–400 µg of protein/mL) in the appropriate culture medium (RPMI 1640 + DMEM with 2% FBS for the A549 cells; serum-free LHC-8 for the BEAS-2B cells) were added to the wells. The cells were then incubated at 37°C for an additional 24 hours. The controls included untreated cells and the medium alone; blank wells without cells were prepared as well. The MTT assay was performed as described in previous studies.^19^ The optical absorbance at 570 nm was measured using a VICTOR X4 Multilabel Plate Reader (Perkin Elmer, Waltham, MA, USA). The results were expressed as (i) the percentage of cell viability relative to the control and (ii) IC50, ie the concentration required to achieve a 50% reduction in cell viability. Each experiment was conducted in triplicate with 4–8 samples per run. The percentage of viable cells was calculated using the following formula:

$${\mathrm{Cell\,vaiability}}\,\left({\mathrm{\% }}\right){\mathrm{=}}{{{\mathrm{A570\,of\,treated\,cells-A570\,of\,blank}}}\over{{\mathrm{A570\,of\,control\,cells-A570\,of\,blank}}}}{\mathrm{\times100}}$$

### Flow Cytometry

#### Cytotoxicity Study

**Figure 1:**
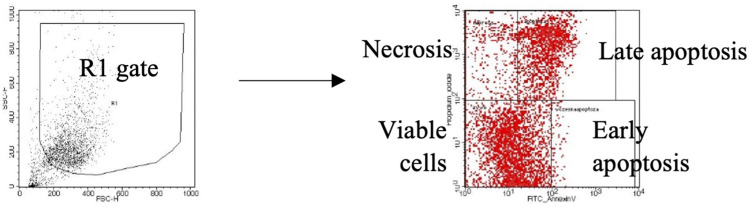
A representative flow cytometry gating strategy for the cytotoxic study.

The cells were seeded into 24-well plates (1 mL per well) at a density of 1x10^5^ cells/mL (the A549 cell line) or 2x10^5^ cells/mL (the BEAS-2B cell line) (Nunc, Roskilde, Denmark) in the appropriate culture medium containing 10% FBS and incubated at 37°C for 24 hours. After removing the medium, fresh medium was added along with the fungal extracts to be tested, with concentrations determined based on the MTT assay results (RPMI 1640 + DMEM with 2% FBS for the A549 cells and serum-free LHC-8 for the BEAS-2B cells). Untreated cells were the negative control, while cells treated with 5% DMSO (Sigma-Aldrich) were the positive control. After 24-hour incubation, supernatants were collected from each well and placed in cytometry tubes. Then, 150 µL of accutase (Corning Media) was added to each well to detach the cells. The contents of each well were resuspended in 500 µL of the respective culture medium (RPMI 1640 + DMEM with 2% FBS for the A549 cells and serum-free LHC-8 for the BEAS-2B cells), transferred to cytometry tubes containing corresponding supernatants, and centrifuged (314 × g, 5 minutes, at room temperature). The supernatants were discarded, and the cells were washed with 500 µL of binding buffer (10X Annexin V Binding Buffer, diluted 1:9 in H_2_O, BD Biosciences). After another round of centrifugation (314 × g, 5 minutes, room temperature), the cell pellet was resuspended in 100 µL of binding buffer with 5 µL of annexin V FITC (BD Biosciences) and 5 µL of propidium iodide (BD Biosciences), gently vortexed, and incubated for 15 minutes at room temperature, protected from light. Finally, 400 µL of binding buffer was added to each cytometry tube, and the samples were analyzed using a flow cytometer (FACSCalibur™ Flow Cytometer, BD Biosciences) with Cell Quest Pro software. Each experiment was performed in triplicate. The results are reported as the percentages of viable, apoptotic (early and late), and necrotic cells among the total cells analyzed. A representative flow cytometry gating strategy for this analysis is presented below (Figure 1).

#### Determination of Reactive Oxygen Species Production

**Figure 2:**
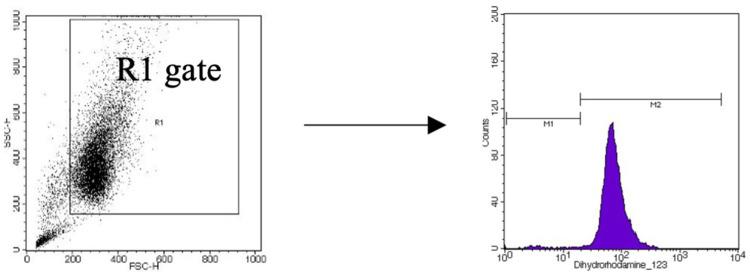
A representative flow cytometry gating strategy for the determination of reactive oxygen species production. M1 –ROS non-producing cells, M2 – ROS producing cells.

Cells from both cell lines were suspended in PBS without Ca^2+^ and Mg^2+^ ions, supplemented with 1% FBS, at a density of 2×10^5^ cells/mL. One milliliter of the cell suspension was placed in a cytometric tube, and 1 µL of dihydrorhodamine 123 (DHR 123, Thermo Fisher Scientific) (final concentration of 5 mm/mL in PBS without Ca^2+^ and Mg^2+^) was added. The mixture was then incubated for 15 minutes at 37°C. The fungal extracts were added to cytometric tubes at doses determined by the MTT assay results. Untreated cells served as a negative control, while cells treated with 5 µL of tert-butyl hydroperoxide (tBHP, Sigma-Aldrich) (final concentration of 50 µM in PBS) were used as a positive control. After 1-hour incubation at 37°C, the tubes were centrifuged (314 × g, 5 minutes, room temperature), and the supernatant was discarded. The cell pellet was resuspended in 500 µL of PBS with 1% FBS, and flow cytometric analysis was performed (FACSCalibur™ Flow Cytometer, BD Biosciences; Cell Quest Pro software). Based on three independent experiment replications, the results were expressed as the percentage of cells producing reactive oxygen species versus those that did not produce ROS. A representative flow cytometry gating strategy for this analysis is shown below (Figure 2).

#### Determination of Cytokine Production

In our experiments, we evaluated the ability of the fungal extract to stimulate the production of IL-1β, IL-6, TNF-α, TGF-β, and GM-CSF in both A549 and BEAS-2B cells. Specifically, cells at a density of 1x10^5^ cells/mL (the A549 cell line) or 2x10^5^ cells/mL (the BEAS-2B cell line) were seeded into 24-well plates (1 mL/well) (Nunc, Roskilde, Denmark) in appropriate culture medium containing 10% FBS and incubated at 37°C for 24 hours. After removing the old medium, fresh medium was added along with the fungal extracts: RPMI 1640 + DMEM with 2% FBS for the A549 cells and serum-free LHC-8 for the BEAS-2B cells. The concentration of the fungal extracts was selected based on the MTT assay results: (i) a concentration equivalent to 50% of the IC50 value and (ii) a concentration where cell viability was within the 80–90% range. Untreated cells served as a negative control. Each experiment was performed in triplicate. After 24 hours of incubation at 37°C, culture supernatants were collected and centrifuged (314 × g, 5 minutes, room temperature). The samples were then stored at −70°C for subsequent analysis. Enzyme-linked immunosorbent assays (ELISA, Shanghai Coon Koon Biotech Co.) were conducted according to the manufacturer’s instructions. The sensitivity of the ELISA tests for each cytokine was as follows: IL-1β 10.0 pg/mL, IL-6 1.0 pg/mL, TNF-α 10.0 pg/mL, TGF-β 10.0 pg/mL, and GM-CSF 10.0 pg/mL.

### Determination of the Presence of E-Cadherin and Occludin With Fluorescence Microscopy

The cells were seeded at a density of 3x10^4^ cells/mL (the A549 cell line) and 6x10^4^ cells/mL (the BEAS-2B cell line) in appropriate culture medium into 8-chamber slides (0.5 mL per chamber) (Thermo Fisher Scientific) and incubated at 37°C for 24 hours. Afterward, the medium containing 10% FBS was replaced with fresh medium containing the fungal extracts at the same concentration range as that used for the cytokine analysis: RPMI 1640 + DMEM with 2% FBS for the A549 cells and serum-free LHC-8 for the BEAS-2B cells. Following another 24-hour incubation at 37°C, the supernatant was removed, and the chambers were rinsed three times with PBS containing Ca^2+^ and Mg^2+^ ions (500 µL per chamber). The cells were then fixed with 4% paraformaldehyde (400 µL per chamber, BD Biosciences) for 15 minutes at 37°C, followed by three washes with 500 µL of PBS. To permeabilize the cell membranes, 400 µL of 0.1% Triton X-100 (Sigma-Aldrich) in PBS was added to each chamber and incubated for 15 minutes at room temperature. After another three washes with PBS, 500 µL of 2% bovine serum albumin (BSA, Sigma-Aldrich) in PBS was added to each chamber to block non-specific antibody-binding sites. The chambers were incubated for 60 minutes at room temperature before the supernatant was removed, and 100 µL per well of primary antibody (occludin - 5 µg/mL in 2% BSA; E-cadherin - 2 µg/mL in 2% BSA) was added (Thermo Fisher Scientific). Following overnight incubation at 4°C, the chambers were rinsed three times with 500 µL of PBS, and 100 µL per well of Alexa Fluor™ 488-conjugated secondary antibody (2 µg/mL in 2% BSA) was added (Thermo Fisher Scientific). The slides were incubated for 45 minutes at room temperature in the dark. After removing the supernatant, 200 µL of DAPI (2 µg/mL in PBS) (Sigma-Aldrich) was added to each chamber and incubated in the dark for 5 minutes at room temperature. The chamber walls were removed, the slides were rinsed with PBS, and a few drops of a glycerol/water solution (1:1, v/v) were applied. The slides were covered with coverslips, and images were captured using a DM4000B epifluorescence microscope (Leica, Wetzlar, Germany) equipped with Leica EL6000 filters, a Leica DFC 500 camera, and LAS V3.1 Leica image analysis software.

Additional control samples were prepared with cells not treated with fungal extracts: (i) cells without primary antibodies and (ii) cells stained only with secondary antibodies conjugated to a fluorescent dye to ensure the specificity of the fluorescent staining.

### Statistical Analysis

Continuous variables with a normal distribution were expressed as mean ± SD, derived from at least three independent experiments. Statistical significance between groups was determined using the Mann–Whitney *U*-test or one-way ANOVA, followed by Tukey’s post-hoc multiple comparison test. Statistical analysis was conducted using STATISTICA version 12 (StatSoft, Inc., Tulsa, OK, USA), with a P value of ≤0.05 considered significant. The IC50 (inhibitory concentration) was calculated using GraphPad Prism version 6 (GraphPad Software Inc., La Jolla, CA, USA).

## Results

### Plant Material and Morphological Identification of Microfungi

**Figure 3:**
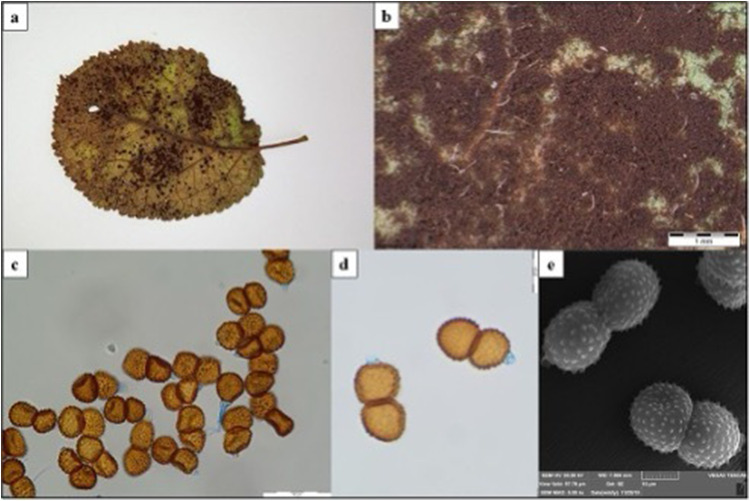
*T. pruni-spinosae* on *Prunus domestica*. (**a**) – disease symptom on infected leaves; (**b**) – telia with teliospores on the lower part of the leaf (SM); (**c** and **d**) – teliospores (LM); (**e**) – teliospores (SEM). Scale: b – 1 mm; c – 50 µm; d – 20 µm; e – 10 µm.

**Figure 4:**
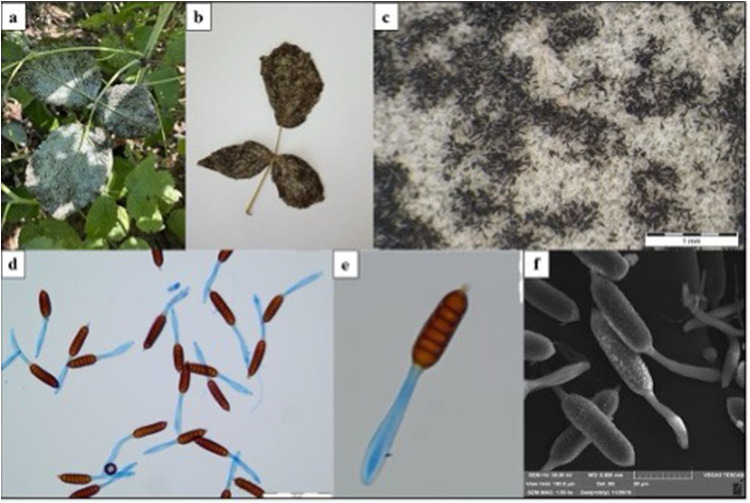
*P. rubi-idaei* on *Rubus idaeus*. (**a** and **b**) – disease symptom on infected leaves; (**c**) – telia with teliospores on the lower part of the leaf (SM); (**d** and **e**) – teliospores (LM); (**f**) – teliospores (SEM). Scale: c – 1 mm; d – 200 µm; e, f – 50 µm.

**Abbreviations**: SM, stereoscopic microscope; LM, light microscope; SEM, scanning electron microscope.

*T. pruni-spinosae* was identified based on the morphological characteristics of the species. Dark brown teliospores of *T. pruni-spinosae* develop on the lower side of *Prunus domestica* leaves (Figure 3a), with corresponding brown spots identified on the upper side (Figure 3b). Teliospores with dimensions of approximately 30–40 × 17–23 µm are structures consisting of two identical cells whose yellow-brown cell wall is covered with papillae regularly distributed over the entire surface (Figure 3c–e). Dark chestnut brown teliospores of *P. rubi-idaei* are located on the lower surface of *Rubus idaeus* leaves (Figure 4a and b). They consist of 5–9 cells with 2–3 germ pores in each cell. The upper part of the teliospores, whose brown cell wall is verrucose, has a yellow, sharp outgrowth up to 11 µm long (Figure 4c). Their dimensions are most often 70–120×27–33 µm. The teliospores are mounted on pedicels that thicken downwards (Figure 4d–f). The morphological features of *T. pruni-spinosae* and *P. rubi-idaei* were consistent with the description of the species.^20^

### Genetic Identification of Microfungal Species and Molecular Phylogeny

Currently, when determining the taxonomic position of fungi, in addition to morphological analysis, information obtained from molecular analyses is also considered.^21^ One of the barcodes commonly used to determine phylogenetic relationships and the taxonomic position is the ITS regions (ITS1 and ITS2). Increasingly, analysis based on several genetic markers is used to determine the taxonomic position of fungal isolates more precisely, such as the LSU sequence, the gene encoding beta-tubulin, or calmodulin.^22^

**Figure 5:**
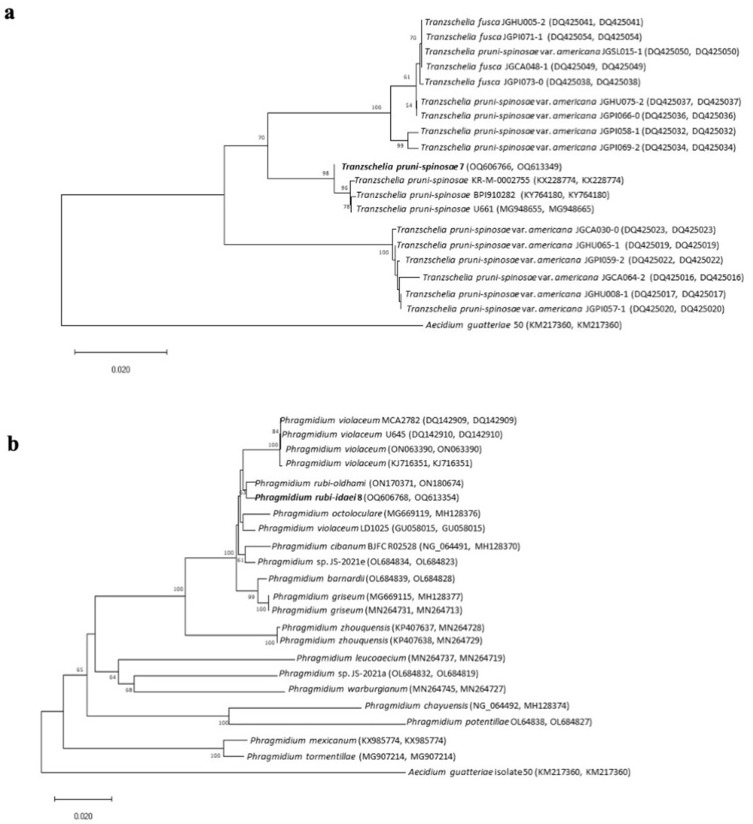
Phylogenetic tree showing the phylogenetic relationships of the *Prunus domestica* (**a**) and *Rubus idaeus* (**b**) isolates and reference strains from the GenBank database, based on sequence similarity of the LSU + ITS regions. Bootstrap values (≥50) are shown on the branches. The scale in the lower left corner indicates the branch length corresponding to 0.02 nucleotide substitutions per base of the compared sequences.

The analysis aimed to establish phylogenetic relationships and determine the taxonomic position of the isolate parasitizing on *Prunus domestica* (domestic plum). Based on the Neighbor-Joining (NJ) method, a phylogram was constructed for the tested sequence (combined sequences of two ribosomal markers: LSU and ITS), showing similarity between the obtained sequences and the reference sequences from the GenBank database (Figure 5a). The analysis showed that the species parasitizing on *Prunus domestica* represents the genus *Tranzschelia*. The degree of similarity of the LSU + ITS sequences to members of this genus ranged from 87% to 99%. The tested isolate showed the highest sequence similarity to strains representing the species *T. pruni-spinosae*, forming a common group with a support rate of 98% (Figure 5a). The results of the morphological analysis combined with the data obtained from the molecular analysis made it possible to assign the tested strain to the species *T. pruni-spinosae*.

Traditional methods used to determine the taxonomic position of the genus *Phragmidium* species are based on teliospore morphology. However, due to the difficulty in distinguishing species solely based on spore morphology, DNA sequence analysis is increasingly included in taxonomic identification and research.^23–25^ To determine the taxonomic position of the isolate from *Rubus idaeus* (raspberry), a phylogenetic analysis was performed based on two loci (LSU and ITS). Comparative analysis of the LSU and ITS region sequences to the corresponding sequences deposited in the GenBank database showed that the isolate represents the genus *Phragmidium*. The degree of similarity of the LSU sequence to the sequence of reference strains of this genus ranged from 88% to 99%, and in the case of ITS, from 69% to 99%. The phylogenetic relationships and the taxonomic position were analyzed using combined LSU and ITS rDNA-based sequence datasets (Figure 5b). The analysis included 23 strains, of which 22 represented the genus *Phragmidium* and one represented the genus *Aecidium*. In the phylogenetic tree, the *Rubus idaeus* isolate formed a common group with the species *P. violaceum, P. rubi-oldhami, P. octoloculare, P. cibanum, P. barnardeii*, and *P. griseum* (with a similarity coefficient of 100%).

### Characteristics of Biological Properties of Tranzschelia Pruni-Spinosae and Phragmidium Rubi-Idaei

#### Cytotoxicity Study (MTT Assay and Flow Cytometry)

**Figure 6:**
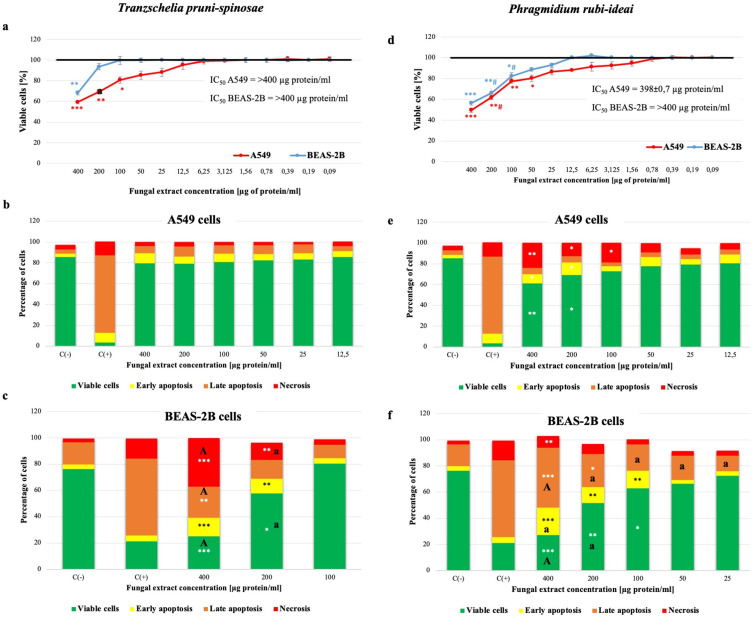
Assessment of the viability of A549 and BEAS-2B cells after 24-hour incubation with *T. pruni-spinosae* and *P. rubi-idaei* fungal extracts (**a** and **d** – MTT method, (**b, c, e**, and **f**) – flow cytometry). The results are mean ± SD values from three independent experiments (in the case of the MTT methods: 4–8 replicates). *Statistically significant difference compared to the negative control sample (Mann–Whitney *U*-test), *P≤0.05; **P≤0.01; ***P≤0.001. ^a^ – statistically significant difference compared to the A549 line cells (the same concentration of the extracts) (one-way ANOVA with Tukey’s post-hoc test), ^a^P≤0.05, ^A^P≤0.001. # - statistically significant difference compared to *T. pruni-spinosae* (Mann–Whitney *U*-test), P≤0.05. C(-) – negative control, C(+) – positive control (DMSO [5%]).

The experiment showed significant cytotoxic activity of the *T. pruni-spinosae* extract only in the highest concentration range of 100–400 µg protein/mL for the A549 cells and 400 µg protein/mL for the BEAS-2B cells (Figure 6a). The level of viable cells after the contact with the extract at the abovementioned concentration was reduced by approximately 19–41% (A549) and 32% (BEAS-2B), compared to the control. A significantly lower percentage of metabolically active alveolar epithelial cells, compared to BEAS-2B, was observed only at the concentration of 200 µg protein/mL (Figure 6a). The IC50 value for the alveolar and bronchial epithelial cells exceeded 400 µg protein/mL. The results of the MTT test showed a toxic effect of the *P. rubi-idaei* extract on the A549 line cells in the concentration range of 50–400 µg of protein/mL - the level of metabolically active cells was lower by approximately 20–50%, compared to that observed in the control (Figure 6d). Moreover, a statistically significant decrease in the viability of the BEAS-2B cells (by approximately 18–44%) was observed after incubation with the tested extract in the concentration range of 100–400 µg of protein/mL (Figure 6d). The IC50 value was 398±0.7 µg protein/mL for the alveolar epithelial cells and >400 µg protein/mL for the bronchial epithelial cells. While the A549 cells were similarly sensitive to both fungal extracts, the BEAS-2B cells were more sensitive to the *P. rubi-ideai* extract than the A549 cells.

Based on the results obtained with the MTT method, only the concentration range of 400–100 µg protein/mL of the *T. pruni-spinosae* extract and 400–25 µg protein/mL of the *P. rubi-ideai* extract were selected for the cytometric analysis of the BEAS-2B cells. The flow cytometric analysis of the A549 cells after the contact with the *T. pruni-spinosae* extract in the concentration range of 12.5–400 µg of protein/mL confirmed the results obtained using the MTT test and did not show significant changes in the subpopulation of viable, early-apoptotic, late-apoptotic, and necrotic cells – their percentage fluctuated within the limits of the negative control value. The stimulation with the highest tested concentration of the fungal extract resulted in a more effective increase in the late apoptotic subpopulation of the BEAS-2B cells than A549 (Figure 6b and c). In the case of the *P. rubi-ideai* extract, the cytometric analysis showed a significant reduction in the percentage of viable (by approximately 16–24%) and an increase in the percentage of early apoptotic (by approximately 9%) alveolar epithelial cells, compared to the control, after the stimulation with the extract at the two highest concentrations, ie, 200 µg of protein/mL and 400 µg protein/mL. Moreover, the percentage of cells that died by necrosis significantly increased from 4.7±0.5% to 18.9±1.2% (100 µg protein/mL), to 12.9±1.2% (200 µg protein/mL), and up to 23.9±1.1% (400 µg protein/mL). The percentage of late apoptotic cell subpopulations in the range of the extract concentrations tested (12.5–400 µg protein/mL) was at the level of the negative control. In response to the *P. rubi-idaei* extract at the 50–400 µg protein/mL concentration, the percentage of both apoptotic and necrotic A549 cells was significantly lower than that of BEAS-2B (Figure 6e and f). The comparison of the toxic effects of the two tested extracts in the concentration range of 200 and 100 µg of protein/mL revealed that the *P. rubi-idaei* extract was significantly more toxic than *T. pruni-spinosae* to the A549 and BEAS-2b cells (Figure 6a–d).

#### Determination of Reactive Oxygen Species Production (Flow Cytometry)

**Figure 7:**
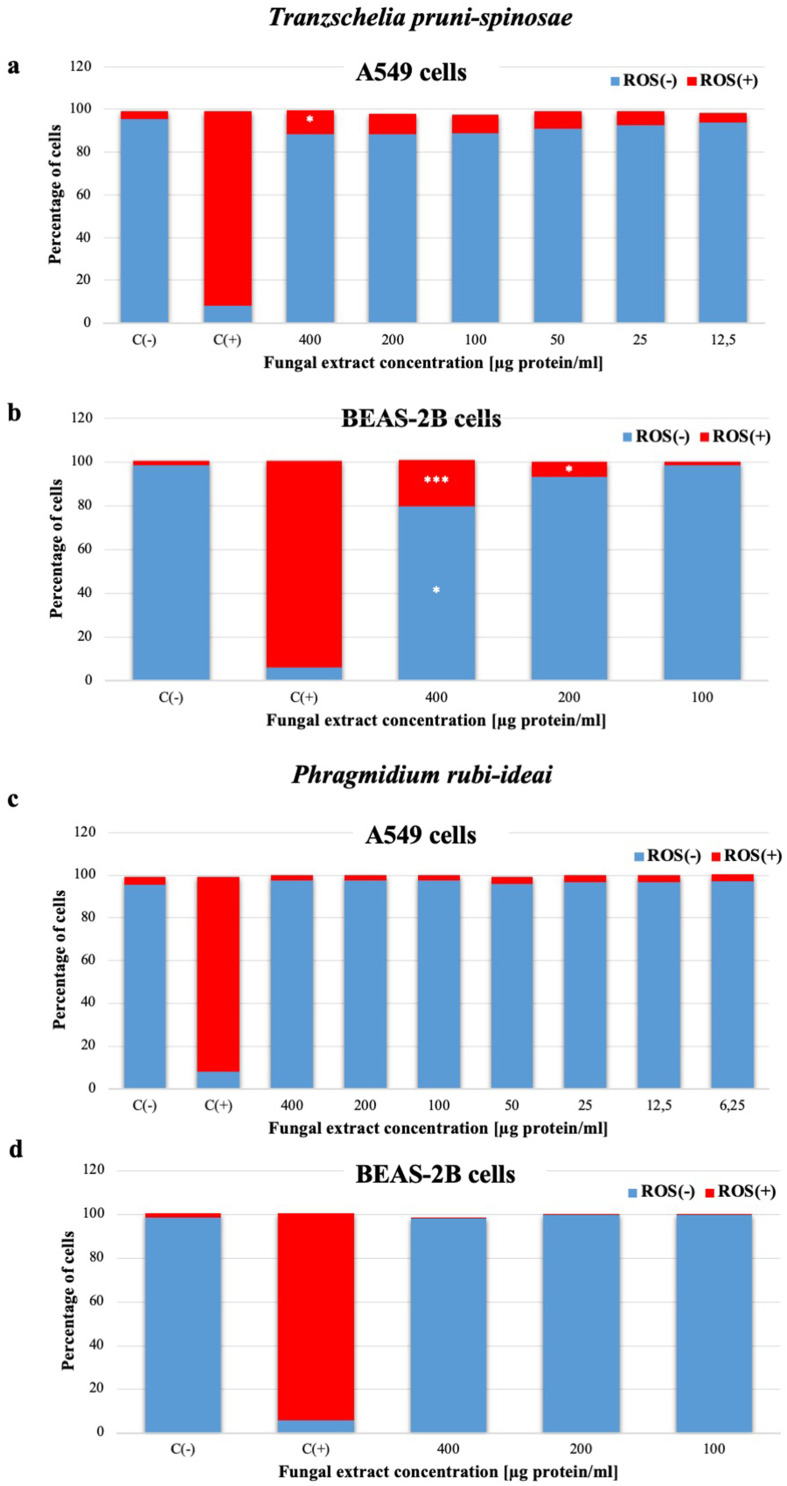
Assessment of ROS production by A549 and BEAS-2B cells after 24-hour incubation with *T. pruni-spinosae* (**a** and **b**) and *P. rubi-idaei* fungal extracts (**c** and **d**). The results are mean ± SD values from three independent experiments. *Statistically significant difference compared to the control sample (C-) (Mann–Whitney *U*-test), *P≤0.05; ***P≤0.001. ^#^ - statistically significant difference compared to *P. rubi-idaei* (Mann–Whitney *U*-test), P≤0.05. C(+) positive control (tBHP [50µM]).

To determine whether reactive oxygen species (ROS) may be the probable cause of the cytotoxic effect of the fungal extract, a cytometric analysis was performed using dihydrorhodamine 123 dye. The percentage of A549 cells generating ROS increased in comparison to the control (3.7±0.2%). It remained at 11.1±1.0% only in response to the highest tested concentration of the fungal extract, ie 400 µg protein/mL (Figure 7a). A similar but statistically significant (approximately 20%) increase in the percentage of cells producing ROS was observed in the case of the BEAS-2B cells treated with the *T. pruni-spinosae* extract at the concentration of 400 µg protein/mL (Figure 7b). Additionally, the ROS level was significantly higher than when the same concentration of the *P. rubi-idaei* extract was used. The experiment showed the inability of the *P. rubi-idaei* extract to induce ROS production in the A549 and BEAS-2B cell lines, regardless of the concentration used (Figure 7c and d).

#### Determination of Cytokine Production

Proinflammatory cytokines with high importance in developing allergic reactions: IL-1β, IL-6, TNF-α, TGF-β, and GM-CSF were selected for the study. Based on the results of cytotoxicity and reactive oxygen production, two concentrations of the *T. pruni-spinosae* extract were used here: 200 and 12.5 µg protein/mL for the A549 line and 200 and 100 µg protein/mL for the BEAS-2B line. In the case of *P. rubi-idaei*, two extract concentrations were chosen: 200 and 6.25 µg protein/mL for the A549 line and 200 and 25 µg protein/mL for the BEAS-2B cells. The level of cytokines was determined after 24-h incubation of the cells with the selected concentrations of the fungal extract.

**Figure 8:**
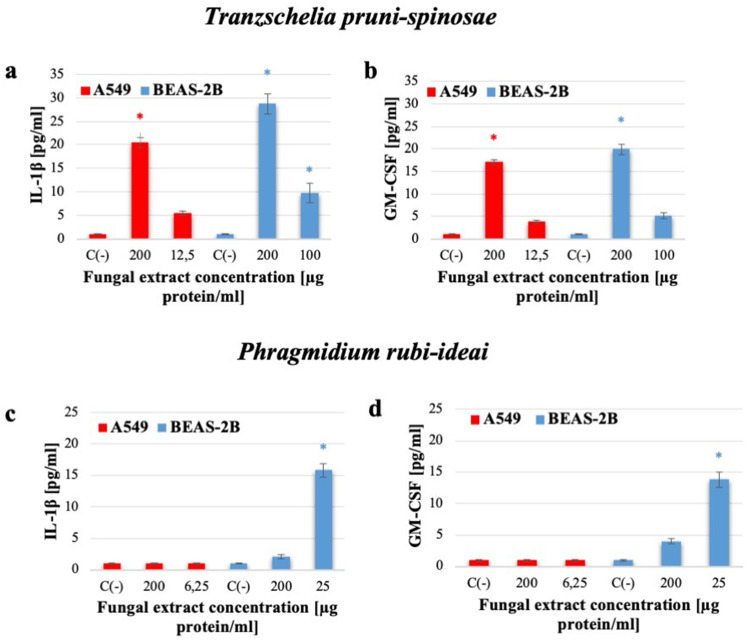
Assessment of IL1-β and GM-CSF production by A549 and BEAS-2B cells after 24-hour incubation with *T. pruni-spinosae* (**a** and **b**) and *P. rubi-idaei* (**c** and **d**) fungal extracts. The results are mean ± SD values from three independent experiments. *Statistically significant difference compared to the control sample (C-) (Mann–Whitney *U*-test), *P≤0.05.

The highest IL-1β level of 28.7±2.2 pg/mL was recorded in the BEAS-2B cells in the treatment with 200 µg protein/mL of the *T. pruni-spinosae* extract (Figure 8a). In the presence of a twice lower extract concentration, the same cells produced IL-1β at a significantly higher concentration (9.8±2.1 pg/mL) than the control cells. A similar relationship was observed in the A549 cells - the concentration of IL-1β in cells stimulated with 200 µg of protein/mL of the extract was significantly higher than its level in cells after the contact with the extract concentration of 12.5 µg of protein/mL (20.5 ±1.1 pg/mL and 5.5±0.5 pg/mL, respectively) (Figure 8a). The *T. pruni-spinosae* extract also significantly stimulated the production of GM-CSF in the cells of both tested lines, but only when the highest concentration of the extract (200 µg of protein/mL) was used (17.1±0.5 pg/mL in the A549 cells and 19 0.9±1.1 pg/mL in the BEAS-2B cells) (Figure 8b). The *P. rubi-idaei* extract induced significant production of IL1-β and GM-CSF in the A549 and BEAS-2 cell lines only at the lower concentration – 25 µg of protein/mL (Figure 8c and d). During the experiment, no IL-6, TNF-α, or TGF-β were released by the A549 and BEAS-2B cells after the stimulation with the tested concentrations of both fungal extracts.

#### Determination of Metalloproteinase 2 and 9 Levels

This study used the same concentrations of *T. pruni-spinosae* and *P. rubi-idaei* extracts to induce proinflammatory cytokines. The level of metalloproteinases was determined after 24 hours of incubation of the cells with the selected concentrations of the fungal extracts.

**Figure 9:**
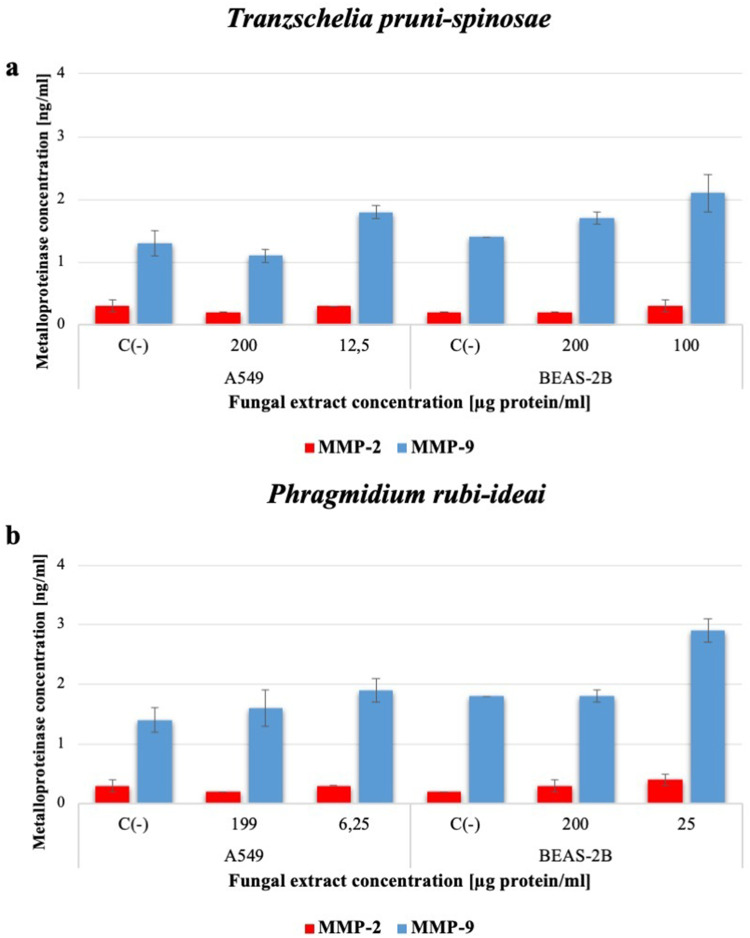
Assessment of MMP-2 and MMP-9 production by A549 and BEAS-2B cells after 24-hour incubation with *T. pruni-spinosae* (**a**) and *P. rubi-idaei* (**b**) fungal extracts. The results are mean ± SD values from three independent experiments. C(-) – control sample. No statistically significant differences were found compared to the control (Mann–Whitney *U*-test).

Both the *T. pruni-spinosae* and *P. rubi-idaei* extracts slightly increased MMP-9 production by the A549 and BEAS-2B cells only at their lower concentrations; however, the differences were not statistically significant. The level of MMP-2 remained practically unchanged, regardless of the fungal species used and the extract concentration (Figure 9a and b).

#### Evaluation of Epithelial Cell Integrity

To determine the presence of intercellular junction proteins E-cadherin and occludin, the same concentrations of the *T. pruni-spinosae* and *P. rubi-idaei* extracts were used to determine cytokine production.

**Figure 10:**
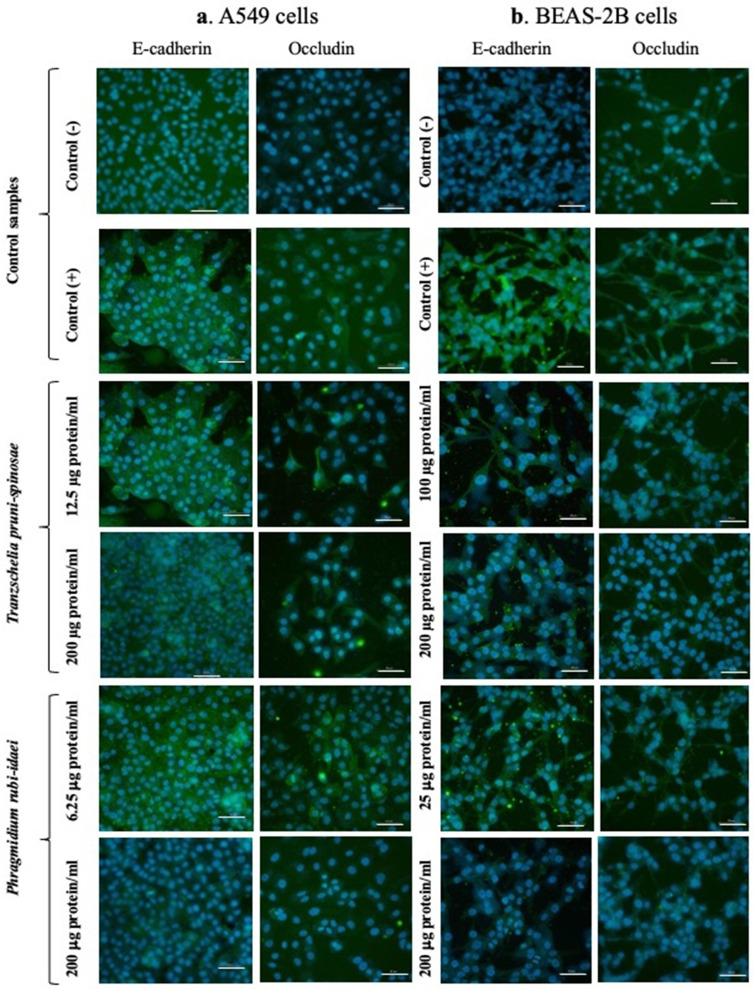
Effect of the *T. pruni-spinosae* and *P. rubi-idaei* extracts on the presence of E-cadherin and occludin in the A549 **(a)** and BEAS-2B **(b)** cells (representative photos from three independent replicates). Control (-) - fungal extract-untreated cells incubated with secondary antibody only; Control (+) - fungal extract-untreated cells incubated with primary and secondary antibody. Cell nuclei stained with DAPI (blue). Bars: 50 µm.

The microscopic observations confirmed the ability of the *T. pruni-spinosae* extract to reduce the fluorescence of E-cadherin and occludin in the A549 cell cultures, which was especially visible at the higher concentration (200 µg protein/mL) (Figure 10a). This may suggest a reduced level of these proteins, weakening cell–cell adhesion. The treatment of the BEAS-2B line cells with the extract resulted in a significant difference in the E-cadherin fluorescence intensity, compared to control cells, with the higher concentration of *T. pruni-spinosae* (200 µg protein/mL) appearing to reduce the level of E-cadherin much more. Similar microscopic observations were noted for occludin (Figure 10b).

After the incubation with the *P. rubi-idaei* extracts at the concentration of 6.25 µg protein/mL, the intensity of E-cadherin fluorescence observed in the culture of the control and A549 cells was similar. However, the exposure of the A549 cells to the higher extract concentration (200 µg protein/mL) reduced the presence of E-cadherin, as evidenced by the lower intensity of green fluorescence than that of the control cells. A similar observation was made in the case of occludin (Figure 10a). Compared to the control cells, the experiment showed a significant inhibiting effect of the *P. rubi-idaei* extract concentrations on the presence of the studied integrity markers in the BEAS-2B cells. The higher concentration of the *P. rubi-idaei* extract (200 µg protein/mL) led to virtually complete disappearance of E-cadherin and occludin fluorescence, which may suggest the absence of both proteins (Figure 10b).

## Discussion

Currently, allergies are being considered an epidemic of the 21st century with a continuously rising incidence. According to the 2013 White Book on Allergy by the World Allergy Organization (WAO), the allergy prevalence ranges between 10% and 40%, depending on the country. Approximately 200–250 million people suffer from food allergies, one-tenth of the population has allergic reactions to medications, and the burden of asthma and allergic rhinitis is estimated at 300 and 400 million people, respectively.^26^ Fungi are known as the most prominent source of allergens. So far, 174 allergens in the phylum Ascomycota and 30 in the phylum Basidiomycota have been described, with the essential allergenic fungi belonging to the genera *Alternaria, Aspergillus, Cladosporium, Penicillium*, and *Fusarium*.^4^

For the first time, we present two phytopathogenic microfungi belonging to the Pucciniales order: *T. pruni-spinosae* and *P. rubi-idaei*. These microfungi exhibit proinflammatory and, thus, allergenic potential. Additionally, to support the microscopic identification of the studied fungal species, we describe their identification at the molecular level.

Based on LSU + ITS sequences, the genetic analyses revealed 69–99% similarity of the microfungal isolates from *Prunus domestica* and *Rubus idaeus* to *T. pruni-spinosae* and *P. rubi-idaei* species.

Due to the small size of fungal spores, which usually does not exceed 10 µm, they can easily penetrate the respiratory tract, including the lungs.^27^ Although the size of *T. pruni-spinosae* and *P. rubi-idaei* teliospores exceeds 10 µm, their penetration into bronchi and alveoli in humans is also probable since even larger fungal hyphae are inhalable.^28^

Like other allergic diseases, asthma is based on inflammatory reactions characterized by the aberrant immune response affecting epithelial cells, fibroblasts, vascular cells, and airway smooth muscle cells, which, in addition to immune cells, also become a crucial source of inflammatory mediators. Some studies have emphasized the importance of epithelial-derived cytokines in promoting Th2 immune responses.^29^ Several studies have identified an essential role of airway epithelial-derived cytokines in asthma pathogenesis, eg proinflammatory IL-1β and IL-6, GM-CSF (recruitment of granulocytes and monocytes), TNF-α (responsible for epithelial integrity), and TGFβ (airway remodeling).^30^,^31^ In our in vitro experiments, we validated the ability of epithelial airway cells to produce proinflammatory cytokines under *T. pruni-spinosae* and *P. rubi-idaei* extract exposure. To determine the proinflammatory and allergenic potential of *T. pruni-spinosae* and *P. rubi-idaei* in the context of airway cells, we used two cell models acceptable in such studies: human normal bronchial epithelial cells (BEAS-2B)^32^ and human lung carcinoma epithelial cells (A549).^32–34^ While the *P. rubi-idaei* extract was an inducer of IL-1β and GM-CSF only in the bronchial epithelial cells (BEAS-2B line), *T. pruni-spinosae* induced these cytokines in both A549 and BEAS-2B cell lines, especially at the higher extract concentrations. In similar experiments conducted with *Erysiphe convolvuli* and *E. palczewskii*, ie microfungi parasitizing common plants *Convolvulus arvensis* and *Caragana arborescens*, we observed their ability to induce proinflammatory cytokines (IL-1β, IL-6, and TNF-α) in bronchial (BEAS-2B line) and alveolar human epithelial cells (A549 line).^35^ However, other authors showed that, in the same BEAS-2B cell line, hyphal fragments of *A. fumigatus* and *P. chrysogenum* increased the expression of IL-1α and TNF-α, but did not induce their release,^36^ whereas A549 cells responded to stimulation with conidia or mycelial fragments of *A. fumigatus* by increased TNF-α and GM-CSF production.^37^,^38^ This may indicate a fungal species-dependent and a cell-dependent inflammatory response.

Airway epithelial cells are the first line of defense against exposure of the airway and lungs to inflammatory stimuli and antigens, and epithelial disruption is one of the characteristics of asthma because it allows inhaled substances to pass more easily into the airway wall to interact with immune and inflammatory cells.^39^ The respiratory tract is often exposed to contact with infectious agents, such as viruses and bacteria, and plant and fungal spores in the atmosphere. All of them may damage the continuity of the respiratory barrier, contributing to the initiation and development of diseases. Tight junctions (TJ) of airway epithelium comprise such proteins as E-cadherin and occludin helping to form the airway’s continuous mechanic barrier. The indirect action of many allergens (mites, pollen, cat, dog, fungal) may lead to decreased levels of these proteins in asthma due to their protease activity^29^,^40^ or ability to induce harmful reactive oxygen species.^41^,^42^

Matrix metalloproteinases (MMPs) are a family of proteolytic enzymes with several critical physiological roles, including remodeling the extracellular matrix, facilitating cell migration, and cleaving cytokines. Most MMPs are not expressed in normal healthy tissues but in inflamed tissues or tissues undergoing repair and remodeling. Pulmonary epithelial cells may also be a significant source of MMPs, as they express MMP‐1, ‐2, ‐7, and ‐9, although their profile may be stimulus-specific.^43–45^ Some evidence indicates the involvement of MMPs in the pathophysiology of fungal infections. As reported by Saadat et al, *A. fumigatus* extract inhibited the production of MMPs by fibrosarcoma cell lines, which was evidenced by gelatin zymography.^46^ In contrast to those studies, the *T. pruni-spinosae* and *P. rubi-idaei* extracts in our experiments only slightly increased of MMP-9 levels in both A549 and BEAS-2B cells, as indicated by ELISA tests.

Many studies have shown that inhalation of different allergens, including fungi, can promote ROS generation.^47^,^48^ For example, *Aspergillus* protease-associated inflammatory response induces mitochondrial ROS production in A549 cells.^49^ Similarly, *A. fumigatus* conidia generated oxygen species in BEAS-2B cells.^50^ From our two fungi extracts studied, only *T. pruni-spinosae* induced ROS production in both A549 and BEAS-2B cells.

Another cause of the disruption of the epithelial barrier is the exposure to inflammatory cytokines. Petecchia et al revealed significantly reduced occludin levels in airway cells after contact with TNF-α, IL-4, and IFN-γ.^51^ A similar role of TNF-α and IFN-γ in increasing airway cell permeability by disrupting claudin-mediated junctions in in vitro studies was described by Capaldo et al.^52^ In human bronchial epithelial cells (HBEC), TNF-α caused loss of occludin and claudins from TJ with redistribution of E-cadherin.^53^ In a recent paper by Sztandera-Tymoczek, *E. palczewskii* and *E. convolvuli* induced marked release of IL-1β and TNF-α, which could also be related to the reduced visibility of E-cadherin and occludin in the A549 and BEAS-2B cells.^35^

In our in vitro experiments, the lower concentrations of the fungal extracts caused a stronger inflammatory effect in most cases. A similar relationship was observed in *P. chrysogenum*, a species whose allergenic properties have been well documented. Oya et al noted that, after bronchial epithelial cells (BEAS-2B) were exposed to various concentrations of *P. chrysogenum* hyphae, the release of IL-1β, TNF-α, IL-6, and IL-1α occurred slightly more intensively at a concentration of 50 μg/mL than at 100 μg/mL.^36^ Kauffman et al observed a similar trend in the A549 cell line. The IL-6 and IL-8 cytokine levels were higher in response to *A. alternata* fungal extract at concentrations of 50, 100, or 200 μg/mL than in response to the highest concentration tested, ie 400 ug/mL. Also, in the *A. fumigatus* fungal extract, cytokine production was most intense at the lowest concentration of 50 μg/mL of fungal extract.^54^

Fungal spores have been recognized as one of the most significant inhalant allergens since the 12th century.^55^ Although the exact incidence of allergy to fungi is not well-defined, it is estimated to range from 3% to 10% of the general population, depending on the area’s climatic conditions.^56^ The best-documented allergenic properties have been reported for common fungal species in the genera *Cladosporium, Penicillium, Aspergillus*, and *Alternaria*.^57^ *Cladosporium herbarum* and *Alternaria alternata* are recognized as the third most common inhalant allergens, following house dust and grass pollen, confirming the need to evaluate the potential risks associated with fungal spores in the atmosphere.^58^ Fungal spores are responsible for IgE-dependent type I hypersensitivity reactions, such as allergic rhinitis and asthma. Their elevated atmospheric concentrations correlate with increased hospitalizations and deaths due to asthma and a higher risk of developing rhinitis.^56^,^59^ Other respiratory clinical syndromes caused by fungi include bronchopulmonary mycoses, allergic sinusitis, and hypersensitivity pneumonitis.^60^,^61^ However, the cause of a pre-existing allergy (allergen) is not always identified through commonly used skin or blood tests, suggesting that other allergens are yet to be discovered. Our research indicates for the first time that microfungi parasitizing common plants such as *Prunus domestica* and *Rubus idaeus* may be a potential source of human allergies, thereby expanding the list of identified fungal allergens.

## Conclusions

In the present in vitro studies, we indicated the potential of *T. pruni-spinosae* and *P. rubi-idaei* extracts to collectively disrupt the epithelial barrier in the upper and lower respiratory tract by induction of proinflammatory cytokines and production of reactive oxygen species and metalloproteinases. Although none of the above parameters was spectacularly high, all of them together could cause a decrease in the levels of TJ proteins, such as E-cadherin and occludin in the epithelial cells.

Hence, it can be speculated that growers may experience allergic reactions when a massive infection of *Prunus domestica* by *T. pruni-spinosae* or *Rubus idaeus* by *P. rubi-idaei* occurs. Our research provides knowledge on new potential respiratory allergens that have yet to be confirmed in in vivo studies.

However, our study has certain limitations. One limitation is that it uses crude microfungal extracts, which require standardization to identify the most active components, including proteins, fatty acids, or their complexes. Another limitation is the need to validate our findings in an animal model. Future research should address these limitations to fully clarify the mechanisms and implications of new fungal allergens in human allergic responses.

## Data Availability

The article contains all the necessary data pertaining to the study. The original findings presented in the research are included in the article. For additional inquiries, please contact the corresponding author.
